# Dynamic disequilibrium-based pathogenicity model in mutated pyrin’s B30.2 domain—Casp1/p20 complex

**DOI:** 10.1186/s43141-022-00300-z

**Published:** 2022-02-21

**Authors:** Alaaeldin G. Fayez, Ghada Nour Eldeen, Waheba A. Zarouk, Khaled Hamed, Abeer Ramadan, Bardees M. Foda, Maha M. Kobesiy, Mai E. Zekrie, Randa S. Lotfy, Mona F. Sokkar, Hala T. El-Bassyouni

**Affiliations:** 1 Molecular Genetics and Enzymology Department, Human Genetics and Genome Research Institute, National Research Centre (NRC), Cairo, Egypt; 2 Clinical Genetics Department, Human Genetics and Genome Research Institute, National Research Center, Cairo, Egypt

**Keywords:** Familial Mediterenean Fever, Pyrin, Casp1, B30.2 domain, In silico analysis

## Abstract

**Background:**

The B30.2 variants lead to most relevant severity forms of familial Mediterranean fever (FMF) manifestations. The B30.2 domain plays a key role in protein-protein interaction (PPI) of pyrin with other apoptosis proteins and in regulation the cascade of inflammatory reactions. Pyrin-casp1 interaction is mainly responsible for the dysregulation of the inflammatory responses in FMF. Lower binding affinity was observed between the mutant B30.2 pyrin and casp1 without the release of the complete pathogenicity mechanism. The aim of this study was to identify the possible effects of the interface pocked residues in B30.2/SPRY-Casp1/p20 complex using molecular mechanics simulation and in silico analysis.

**Results:**

It was found that Lys671Met, Ser703Ile, and Ala744Ser variants led mainly to shift of the binding affinity (∆G), dissociation constant (K_d_), and root mean square deviation (RMSD) in B30.2/SPRY-Casp1/p20 complex leading to dynamic disequilibrium of the p20-B30.2/SPRY complex toward its complex form. The current pathogenicity model and its predicted implementation in the relevant colchicine dosage were delineated.

**Conclusion:**

The molecular mechanics analysis of B30.2/SPRY-p20 complex harboring Lys671Met, Ser703Ile, and Ala744Ser variants showed dynamic disequilibrium of B30.2/SPRY-casp1/p20complex in context of the studied variants that could be a new computational model for FMF pathogenicity. This study also highlighted the specific biochemical markers that could be useful to adjust the colchicine dose in FMF patients.

## Background

Familial Mediterranean fever (FMF) is an autosomal recessive periodic fever disorder that is characterized by recurrent unprovoked attacks of fever and serositis including peritonitis, pleuritis, and arthritis. It mostly occurs in populations of Mediterranean descent and runs within families [1]. Familial Mediterranean fever (FMF) is caused by the dysfunction in pyrin, which is encoded by the *MEFV* gene located on chromosome 16 [2].

Human pyrin protein is expressed mainly in the innate immune system cells. Pyrin protein analysis revealed that it consists of five domains, the N-terminal PYD domain (1-92), bZIP transcription factor domain (266-280), the B-box (370–412), α-helical coiled-coil (420–440) domain, and the C-terminal B30.2/rfp/PRY/SPRY domain (597-776) [3].

The B30.2 domain was first identified as a protein domain encoded by an exon (named B30-2) in the Homo sapiens class I major histocompatibility complex region [4]. It consists of PRY and SPRY subdomains. The ~200-residue B30.2/SPRY (for B30.2 and/or SPRY) domain is present in many proteins with various functions in different cellular pathways through protein-protein interaction [5].

The B30.2/SPRY domain assumes a highly distorted, compact beta-sandwich fold with two additional short α-helices at the N-terminus. Both the N- and C-terminal ends of the B30.2/SPRY domain are close to each other. This structural feature and the monomeric nature suggest that B30.2/SPRY domains in general are suitable for serving as a modular domain in multi-domain proteins [6]. The β-sandwich of the B30.2/SPRY domain consists of two layers of β-sheets: sheet A is composed of eight strands (β1, β4, β6, β7, β8, β9, β10, and β14) while sheet B is comprised of seven strands (β2, β3, β5, β11, β12, β13, and β15)_._

The interactions between α1 and the β-sheet are extensive and exclusively hydrophobic, indicating that the helix is structurally important probably for stabilizing the curvature of the highly distorted β-sandwich. The short two-turn helix α 2 interacts with a part of one edge of the β-sandwich. The interaction between the two involves many water molecules and is predominantly hydrophilic, indicating that the presence of this helix may not be absolutely required for the β-sandwich scaffolding [7]_._

In the pyrin protein, the B30.2/rfp/PRY/SPRY domain-mediated protein interactions indicate the role of this domain as an adaptor module to assemble macromolecular complexes [8].

Most of the *MEFV*-associated missense variants hot-spots are clustered within the C-terminal B30.2 domain, and thus, it is crucial for the molecular cascades leading to FMF [9].

In the present study, we aimed to postulate the possible pathogenicity cascade of the studied B30.2 domain variants to extend our previous published work of Zarouk et al. [10]. Applied bioinformatics tools, based on protein structure, alternative splicing, and molecular mechanics simulation were implemented. To the best of our knowledge, this is the first study to use the molecular mechanics computations to identify the relevant pathogenicity of the FMF variants, explore most of the prospective registered interacted proteins, and provide support for physicians in decision-making regarding the selectivity of colchicine administration to the specific B30.2 variants.

## Methods

### Data retrieving

Based on our previous published literature [10], we proceeded with the classification of B30.2/SPRY (580-775aa) variants to test its implications on FMF pathogenicity using different computational biology algorithms and molecular mechanic’s study. The 3D of the B30.2/SPRY domain was retrieved from PDB [11] under code 2wl1 (x-RAY; 1.35A, 586-776aa). 3D of registered relevant Pyrin reactants (CASP1, ULK1 LIR motif bound to GABARAP, ASC, RHOA with kinase pkn/prk1, LRR domain of NLRP1, CARD domain of NLRP1, NLRP3, and IL-18) were attained under codes 1rwp, 6hyo, 2kn6, 1cxz, 4im6, 4ifp, 2naq, and 3wo2 respectively, and immature IL-1 beta protein was modeled by Phyre2 server because its PDB crystallography images is not registered.

It worth to mention that 2wl1 PDB image of the B30.2/SPRY domain (1. 35A°) is the best resolution X-ray image for this domain registered till now.

Computated interactive interface and interaction of atoms between B30.2/SPRY and its reactants; superimposed fitting and interaction residues detection were done using Swiss pdb viewer [12], discovery studio visualization softwares [13], and template interface architecture-dependant PRISM server [14].

Calculated chemical and physical properties of pyrin; differentially polarity and hydrophobicity of the retrieved variants were calculated by ProtoScale (https://www.web.expasy.org/protoscale). Alternative splicing events and scores were computated by MutationTaster (MT) [15] and NNsplice software (www.fruitfly.org/seq_tools/splice).

Molecular mechanics of B30.2/SPRY-p20 complex either wild B30.2/SPRY or mutant B30.2/SPRY were calculated by Swiss Pdb viewer [12], PRISM [14], SAAMBE-3D [16], and PRODIGY [17] servers.

## Results

### The retrieved B30.2/SPRY variants were classified according to its corresponding physicochemical and electrostatic properties

**Fig. 1:**
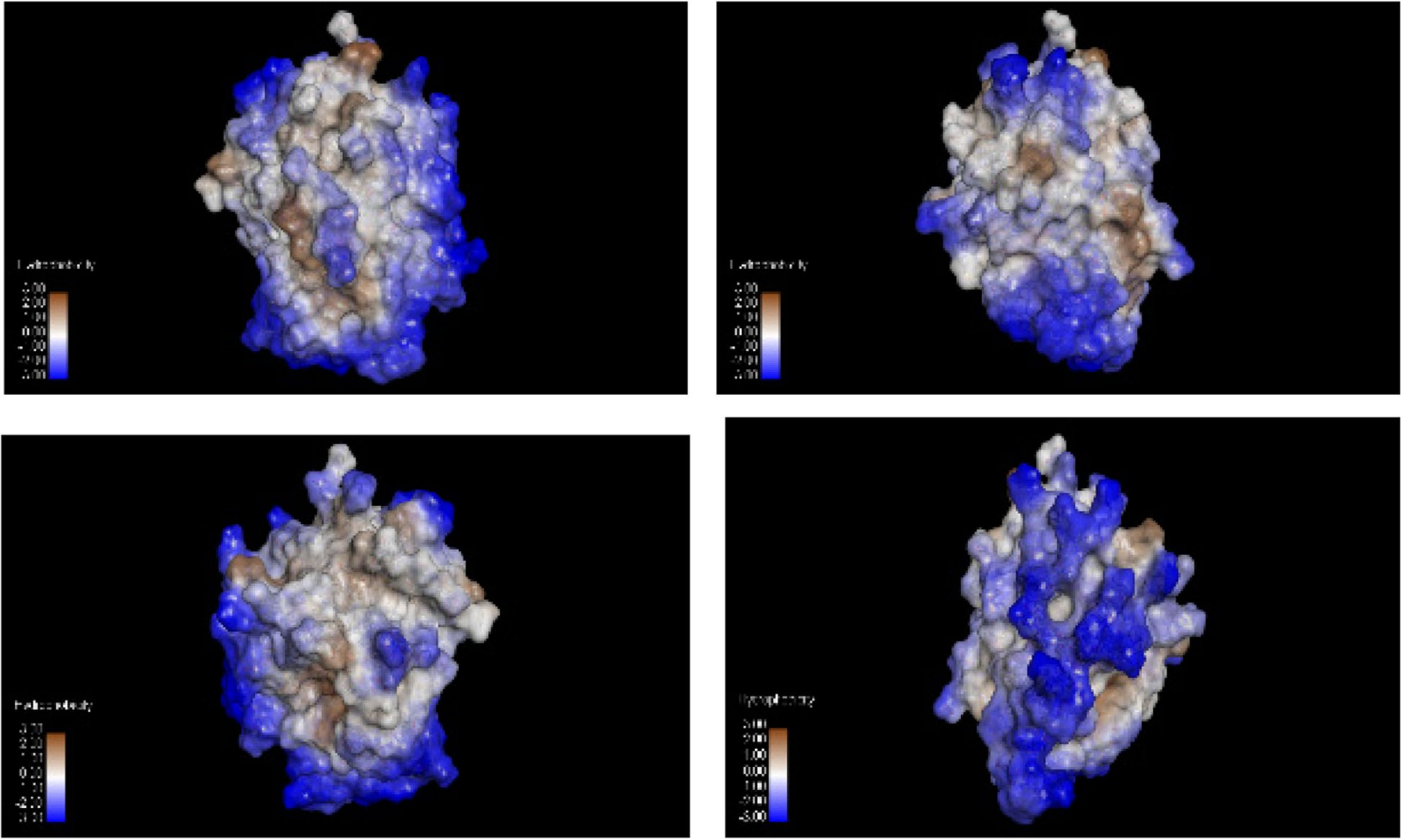
Hydrophobic mapping of b30.2/SPRY domain was done by SWISS-pdbViewer, 90° rotation difference between each image and the other was designed. According to Kyte and Doolittle [18] hydrophobicity scale, residues with a hydrophobicity of 0.7 or more are hydrophobic and those under −2.4 are hydrophilic, so the brown and light brown highlighted spots are hydrophobic patches, the blue highlighted spots are hydrophilic, and the light spots are neutral.

**Fig. 2:**
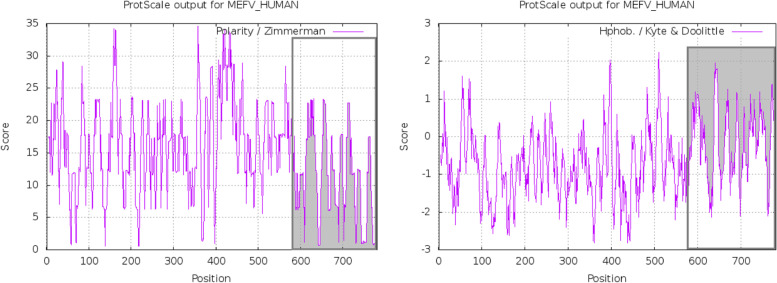
Weighted scores of full-length pyrin residues demonstrate that B30.2/SPRY residues, highlighted by the gray signal, are more to be classified as non-polar hydrophobic residues according to the ProtScale analysis

Doubtless that the 3D of B30.2/SPRY domain of pyrin gave a better base line to construct the relation between the molecular mechanics (MM), location and function of each amino acid, and its mutant pattern implications. This B30.2/SPRY domain (580-775 aa) consists of a α 5-strand and 7-strand β sandwich forming the core of the domain with lots of outer 21 hydrophobic patches, 6 cavities (Area ≥55A°^2^ volume ≥31A°^3^), and 12 wide grooves (Area ≥107A°^2^ volume ≥49A°^3^). These hydrophobic patches (as shown in Fig. 1) have significant binding sits on B30.2/SPRY domain surface, where this domain fragment is a non-polar hydrophobic residue rich out of the full length of pyrin protein as shown in Fig. 2.

**Fig. 3:**
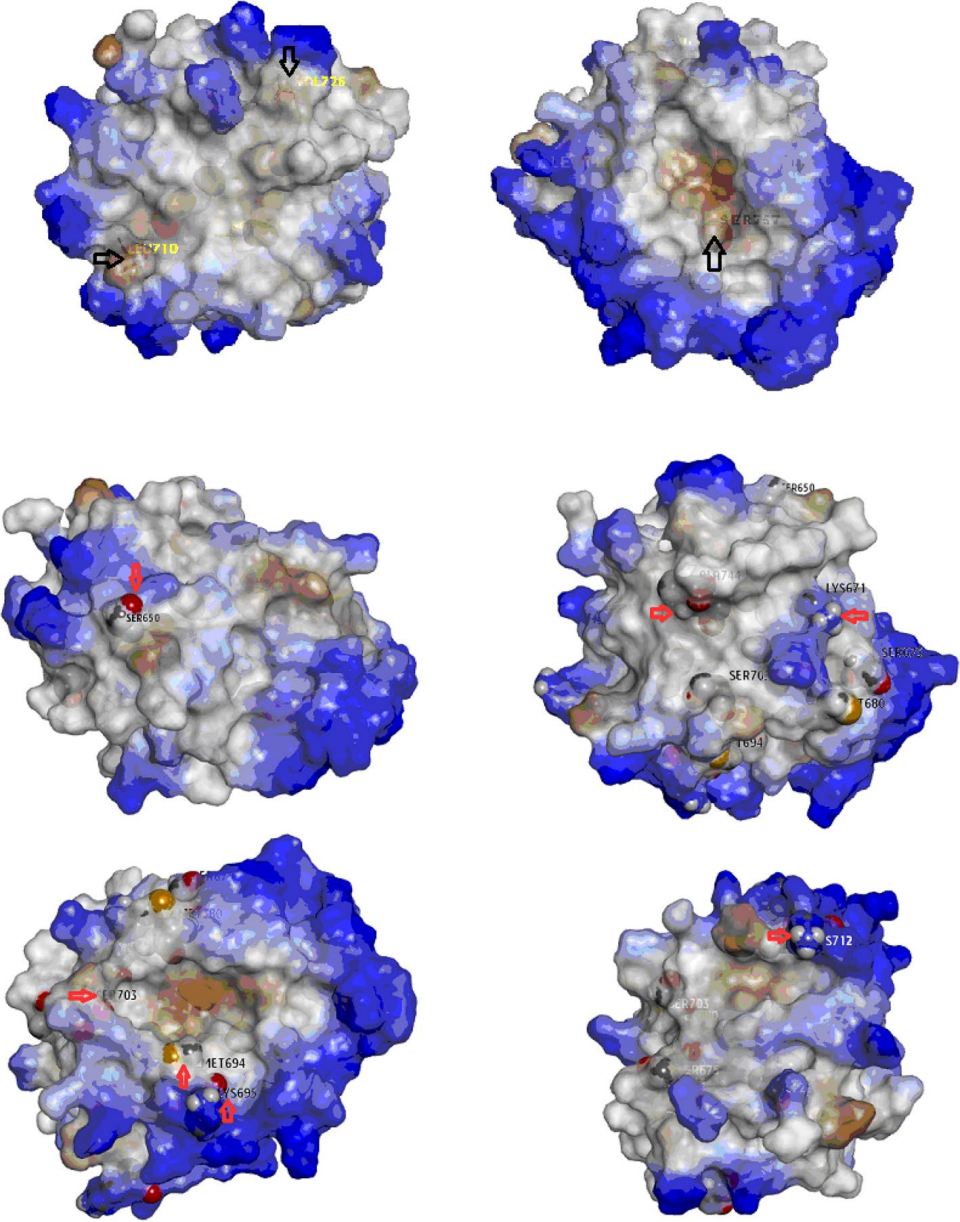
Localization of the 13 retrieved B30.2 domain variants on the hydrophobicity mapping, the arrows point to each variant localization

Annotation of the 13 retrieved variants against the hydrophobicity mapping of the crystallographic model of B30.2/SPRY domain (PDB ID 2wl1) reported that; Out of the 13 variants, S757 and I720 are associated with the detected cavities. While L710, V726, S757, K671, and S695 are associated with hydrophobic patches, whereas V726, M680, M694, S703, S757, and L710 are associated with wide grooves. Accordingly, based on the hydrophobic map and geographic location, the retrieved variants might be classified to (I) L710, V726, A744, and S757 associated with wide hydrophobic grooves, so its properties are prone to be active binding sites; however, S650, K671, S703, M694, S695, and K712 are polar and basic residues (except M694) localized on the edge of the hydrophobic cavities, so it might be considered as active recognition sites as stabilizer for B30.2/SPRY reactants, shown in Fig. 3. On the other hand, each of M680, I720, and S675 could not be classified.

### Interface pocket atoms of B30.2/SPRY-Casp1 inflammosome complex: molecular modulation

**Fig. 4:**
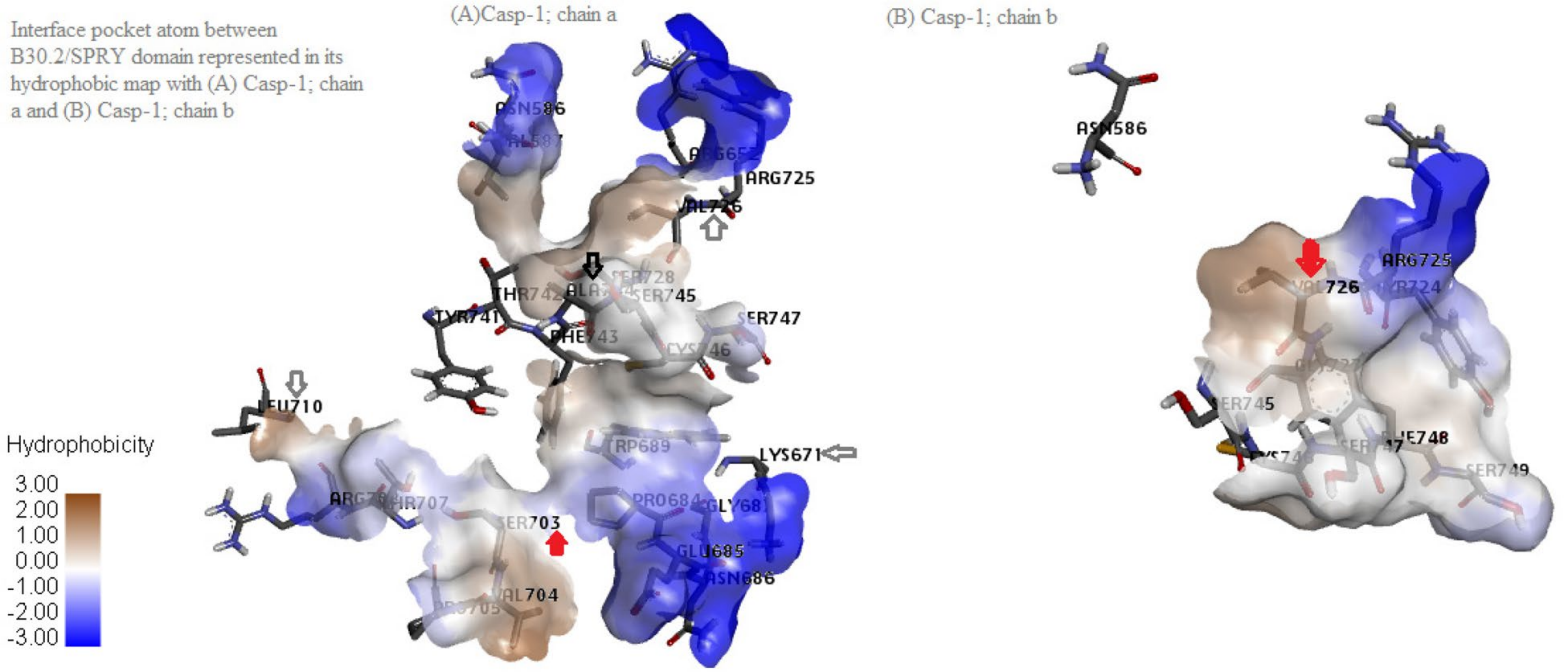
Interface pocket amino acids between B30.2/SPRY and both p20 (**A**) and p10 (**B**) subunits of Casp-1 protein. The contributed amino acids inside the interface pocket divided to interacted residues (non-covalent bonding residues) and nearby ones (bonding scale <5 A° between its Cα atom and that of a surface residue as default setting). Out of the 13 retrieved variants, the interface nearby residues were arrowed by non-filled arrows, and the interacted ones were arrowed by the red filled arrows (B30.2/SPRY:Ser703 with casp1-p20:Ser175 ) & (casp1-p20:Val338 - B30.2/SPRY:Val726. CHARMM52 force field was applied and implemented in the prism server

Casp-1 protein consists of binding two heterodimers, subunits p20 and p10. Prism server predicts the binding residues of protein-protein interaction by using structural similarity and evolutionary conservation of the putative hot spot binding residues. Based on flexible induced-fit backbone refinement in molecular docking by fiberdock implemented in prism server to find optimum combination of rotamers with the lowest total energy, the predicted interaction model between B30.2/SPRY domain of pyrin (PDB id 2wl1) and Casp-1 (PDB id 1rwp with chain a [p20] and b [p10]) was generated. The lowest global energy was selected as the almost stable complex, energy minimization was done by GROMOS96 the implementation of SWISS-pdbViewer, water and ligands were removed, polar hydrogen atoms were added to stimulate the reliable conditions, and the default implemented interaction criteria in the prism included maximum distance of strong hydrogen bond = 3.4 A°, maximum distance of weak hydrogen bond= 3.8 A°, and maximum distance of salt bridge bond = 4 A°. The interface pocket atom of B30.2/SPRY with both p20 and p10 subunits of Casp-1 has not any unfavorable bonds, so its stability is preferred. All the interacted residues were listed in Table 1. Although both subunits p20 and p10 contact with B30.2/SPRY domain, but we found that the surface contact area of p20 was bigger than the surface contact area of p10 (as shown in Fig. 4). Therefore, we hypothesized that the stability of B30.2/SPRY-Casp1 complex is largely due to p20. To clarify this hypothesis, we measured the differential binding affinity (∆G) and dissociation constant (K_d_) between them.

**Table 1:** All the modeled interacted residues between B30.2/SPRY, p20, and p10 of Casp-1

Interacted residues	Distance (A°)	Bonding type
*With p20 (surface area=749.115A°-volum=23.979A*^*3*^*)*		
PRYSPRY: ASN586:H1 - CASP A: ASP288:OD1	2.18794	Hydrogen bond; electrostatic
CASP A: PHE173:H - PRYSPRY: GLU685:OE2	1.90679	Hydrogen bond
CASP A: SER175: HG - PRYSPRY: VAL704:O	2.28906	Hydrogen bond
PRYSPRY: VAL704:H - CASP A:ASP174:O	2.07959	Hydrogen bond
PRYSPRY: SER745: HG - CASP A: GLY238:O	1.81599	Hydrogen bond
CASP A: ARG178:CD - PRYSPRY: GLU685:O	3.03911	Hydrogen bond
PRYSPRY: SER703:CB - CASP A:SER175:O	3.13259	Hydrogen bond
CASP A:PRO177 - PRYSPRY:PRO684	4.93898	Hydrophobic
PRYSPRY: ARG725 - CASP A:PRO290	5.10875	Hydrophobic
PRYSPRY: CYS746 - CASP A:PRO177	3.92809	Hydrophobic
PRYSPRY: TRP689 - CASP A: PRO177	4.7086	Hydrophobic
*With p10*		
B: VAL338 - A: VAL726	4.46634	Hydrophobic

### According to the binding affinity (∆G) and dissociation constant (K_d_) of B30.2/SPRY-Casp1: mutant B30.2/SPRY heightened the binding affinity and decrease the corresponding K_d_

Molecular mechanics of the interface atoms of B30.2/SPRY-Casp1 complex were computed using discovery studio visualizer, swiss-pdbViewer, SAAMBE-3D, and PRODIGY servers. By the discovery studio visualizer and swiss-pdbViewer, we computed both solvent accessibility (SAS) and root mean square deviation (RMSD) of wild and mutant interfaced atoms, PRODIGY server to compute ∆G, and Kd and SAAMBE-3D to determine the effect of mutants on binding energy scores and complex stability. The minimized energy and polar hydrogen addition for 3D of B30.2/SPRY-Casp1 complex were used after removing all the water molecules, small ligands, and applying simulated temperature at 25°C under neutral pH condition.

Through the molecular mechanics (MM) computations of the retrieved variants which included in interface pocket atom of B30.2/SPRY-Casp/p20 complex, we found that mutant Ile703, Ser744, and its combined patterns increased the binding affinity and strength between p20 subunit and B30.2/SPRY domain according to its corresponding ∆G and *Kd* values (Table 2). While Ile 703 variant led to the destruction of the hydrogen bond with p20:Ser175, but increasing of the molar concentration of the complex versus reactants resulted in a dynamic disequilibrium state towards p20-B30.2/SPRY complex formation.

**Table 2:** Molecular mechanic properties of the retrieved variants included in p20-B30.2/SPRY complex

Variants	Clinical pathogenicity	IT.	Binding affinity ∆G ∆∆G Kcal/mol		Kd [M] ∆Kd		HYD	SAS Unbound		∆SAS		RMSD (A°)/∆RMSD
								SASr	SASs	SASr	SASs	
**WILD (ref.)**			−9.5		1E−7							***Ref.=118.493***
Lys671 (W)	Novel	nearby					−3.9	54.9	52.9	−2.1	−3.2	
Met671 (M)			−9.7↑	−0.2	8.2E−8↓	−1.8E−8	1.9↑↑	52.8	49.7			
Ser703 (W)	Novel	int.					−0.8	37.3	27.5	16.8	20.3	
Ile703 (M)			−10.2↑↑	−0.7	3.4E−8↓↓	−6.6E−8	4.5↑↑	54.1↑↑	47.8↑↑			144.072/**25.579** ↑↑
Leu710 (W)	Novel	nearby					3.8	142.3	120.7	−30.6	−40.7	
Pro710 (M)			−9.3↓	0.2	1.5E−7↑	0.5E−7	−1.6↓↓	111.7↓↓	80.0↓↓			118.458/−**0.035**
Val726 (W)	Pathogenic	nearby					4.2	84.4	70.9	−26.3	−33.2	
Ala726 (M)			−9.3↓	0.2	1.5E−7↑	0.5E−7	1.8↓	58.1↓	37.7↓↓			118.470/−**0.023**
Ala744 (W)	Likely	nearby					1.8	66.6	38.2	14.5	18.7	
Ser744 (M)	pathogenic		−10.3↑↑	−0.8	2.7E−8↓↓	−7.3E−8	−0.8↓	81.1↑	56.9↑			118.509/**0.016**
Mut. pattern												
744+726			−10.1↑↑	−0.6	3.9E−8↓↓	−6.1E−8						118.489/−**0.004**
+710			−10.1↑↑	−0.6	3.9E−8↓↓	−6.1E−8						118.458/−**0.035**
+703			−10.7↑↑	−1.2	1.3E−8↓↓	−8.7E−8						118.499/**0.006**
+671			−10.9↑↑	−1.4	1.1E−8↓↓	−8.9E−8						

***∆Kd*** [mKd]-[wKd] molar; *SAS* scores measure the solvent accessibility surface of residues; ***∆SAS*** SASm-SASw where *m* refers to the homozygous mutant and *w* refers to homozygous the wild pattern of variant; *HYD* is sequence-based hydrophobicity scores according to Kyte and Doolittle hydrophobicity values; 0.7 or more are hydrophobic and those under −2.4 are hydrophilic; *∆RMSD* mRMSD-refRMSD concerns the distances between C^α^, main chain, side chain and all heavy metals; *SASr* refers to the solvent accessibility of the main backbone; *SASs* to solvent accessibility of side chain of residues; criteria of SAS computation are for pocket atoms only/probe radius=1.4A° equal to water molecule/grid point per atom=240 implemented in discovery studio visualization tool

↑ mild increase

↑↑ marked increase

↓ mild decrease

↓↓ marked decrease

The next question was which non-bonds force causes that? To investigate this query we calculated the non-bonding energy, solvent accessibility surface (SAS), root mean square deviation (RMSD), and hydrophobicity score of Ile703, Ser744 to find that the substitution of serine by Isoleucine at codon 703 led to the increase of the hydrophobicity at this codon location and increased the solvent accessibility surface from 27.5 to 47.8 and RMSD was increased by 25.579 A°. On the contrast, the substitution of alanine by serine in codon 744 led to the decrease of the hydrophobicity and the increase of the solvent accessibility surface from 38.2 to 56.9 with approximately constant RMSD. Therefore, the non-bond force behind Ile703 dynamic disequilibrium was the hydrophobic interaction and the one behind ser744 might be due to polar-polar interaction.

It is worth to note that despite both Ile703 and Ser744 variants led to increased solvent accessibility surface, the expected hidden force to increase its binding affinity was different. The increased solvent accessibility surface of Ser744 combined with stable RMSD against increased solvent accessibility surface of Ile703 combined with increased RMSD refer to that the effect of high SAS might be abolished by the high distance measured by RMSD.

Although all Met671, Pro710, and Ala726 variants had a mild change in ∆G, this caused considerable change in *Kd*. Met671 led to decrease in the dissociation constant while both Pro710 and Ala726 variants resulted in slight increase in the dissociation constant. According to the MM scores of these variants, we found that both Pro710 and Ala726 variants induced decrease in its hydrophobicity nature and SAS created a new unfavorable bond and hence increased the corresponding *Kd*, in contrast Met671 induced remarkable increase in its hydrophobicity nature from −3.9 to 1.9 and created a new hydrophobic bond combined with slight increasing in ∆G.

To investigate this hypothesis of the creation of a new unfavorable bond that led to the increase in the dissociation constant of Pro710 & Ala726 variants—containing complex, we measured the angle and torsion energy of the wild and mutant patterns and found that Pro710 led to the increase of angle energy from 2.6 to 17.6 and torsion one from 9.4 to 17.4 (data are not tabulated here).

Finally, irrespective of the pathogenicity mechanism of Ser744, Pro710, Ile703, Ala726, and Met671, the current MM scores prone to classified it as severity relevant variants.

### Molecular dynamics scores are molecularly refined hypothesis ASC-independent on NF-kB activation pathway in FMF patients: dynamic disequilibrium phenomenon

According to the available literature, how the pyrin affects NF-kB activation and whether ASC is dependent or independent on NF-kB activation remain unclear. The independent manner concludes that pyrin harboring variants in B30.2/SPRY domain induced increase in the affinity to casp-1 subunits and consequently increase of cleavage probability of pyrin by Casp-1 at Asp330, liberating excess of a 330-residue N-terminal fragment (N330) that activates the NF-kB directly.

Although the mutant B30.2/SPRY domain is more binding with Casp-1 subunits than the wild B30.2/SPRY, the interpretation of this differential binding at the molecular level has not been previously reported. The current results enforce the hyperactivation of the ASC-independent NF-kB in FMF patients and give its molecular-based mechanism of action according to the dynamic disequilibrium state resulting from Lys671Met, Ser703Ile, and Ala744Ser variants.

Dynamic equilibrium occurs when the rate of formation or dissociation of the target complex is in a balance rate with its reactants, consequently when factors disrupt this balance, this may either led to association or dissociation of the target complex.

The molecular mechanics (MM) scores of B30.2/SPRY-Casp1/p20 complex showed that Lys671Met, Ser703Ile, and Ala744Ser variants led to lower dissociation constant (Kd) than wild B30.2/SPRY ones, and hence these variants might lead to dynamic disequilibrium toward the B30.2/SPRY-Casp1 complex as illustrated in equation (1, a & b).

- ***In the wild B30.2/SPRY (dynamic equilibrium)***

$$\left[{\mathrm{P}\mathrm{yrin}}^{\mathrm{B}30.2/\mathrm{SPRY}}\right]\mathrm{w}\left[\mathrm{Casp}{1}^{\mathrm{P}20}\right]\rightleftharpoons \left[\mathrm{B}30.2/\mathrm{SPRY}\hbox{-} \mathrm{P}20\right]$$

(b) ***In the mutant B30.2/SPRY (dynamic disequilibrium)***

$$\left[{\mathrm{P}\mathrm{yrin}}^{\mathrm{B}30.2/\mathrm{SPRY}}\right]\mathrm{m}\;\left[\mathrm{Casp}{1}^{\mathrm{P}20}\right]\rightleftharpoons \left[\mathrm{B}30.2/\mathrm{SPRY}\hbox{-} \mathrm{P}20\right]$$

where *w* refers to wild and *m* refers to mutant

### Relevance of the current dynamic disequilibrium phenomenon to colchicine dosage for FMF patients

IkB-α is a nuclear localization repressor during its binding with NF-kB transcription factor preventing the entrance of NF-kB inside the nucleus and hence abolishes NF-kB to target the corresponding regulatory DNA sequence elements [19].

Although the mechanism of action of colchicine is under discussion, it is still considered as a standard treatment for FMF. It was found that co-transfected cells treated with colchicine led to the decrease of IkB-α proteolysis in a dose-dependent manner, where minimal concentrations of colchicine were effective for the inhibition of the proteolysis of IkB-α [20].

Consequently, the refinement of ASC is independent on the activation pathway of NF-kB and colchicine as an inhibitor to IkB-α proteolysis. In view of the current relevant molecular dynamic results, it led us to predict that in case of the FMF patients harboring Lys671Met, Ser703Ile, and Ala744Ser variants, hyperactivation of NF-kB and increased IkB-α proteolysis might occur and hence the colchicine treatment is preferred to higher its doses than patients with negative Lys671Met, Ser703Ile, and Ala744Ser.

**Fig. 5:**
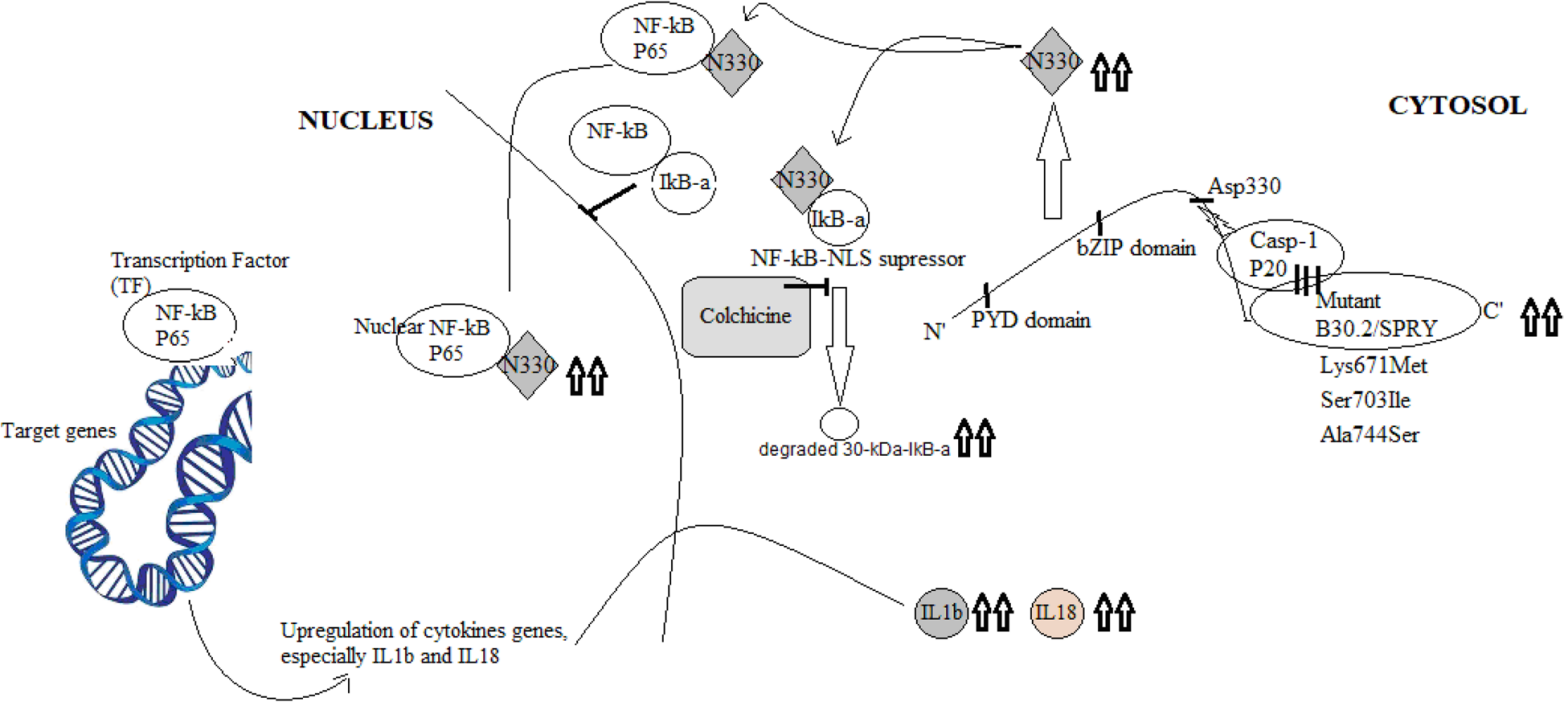
Remodeling of ASC-independent NF-kB activation pathway (modified predictive model) according to our molecular mechanics scores of mutant B30.2/SPRY domain. The three upright lines between casp-1/p20 and B30.2/SPRY mean high binding affinity, while the upright arrows mean elevated quantity, and the bold truncated line means inhibitory effect

The modified predicted model is showed in Fig. 5.

### S757T, K695R, M680I, and S273A might cause dominant faulty of isomers mRNA

**Fig. 6:**
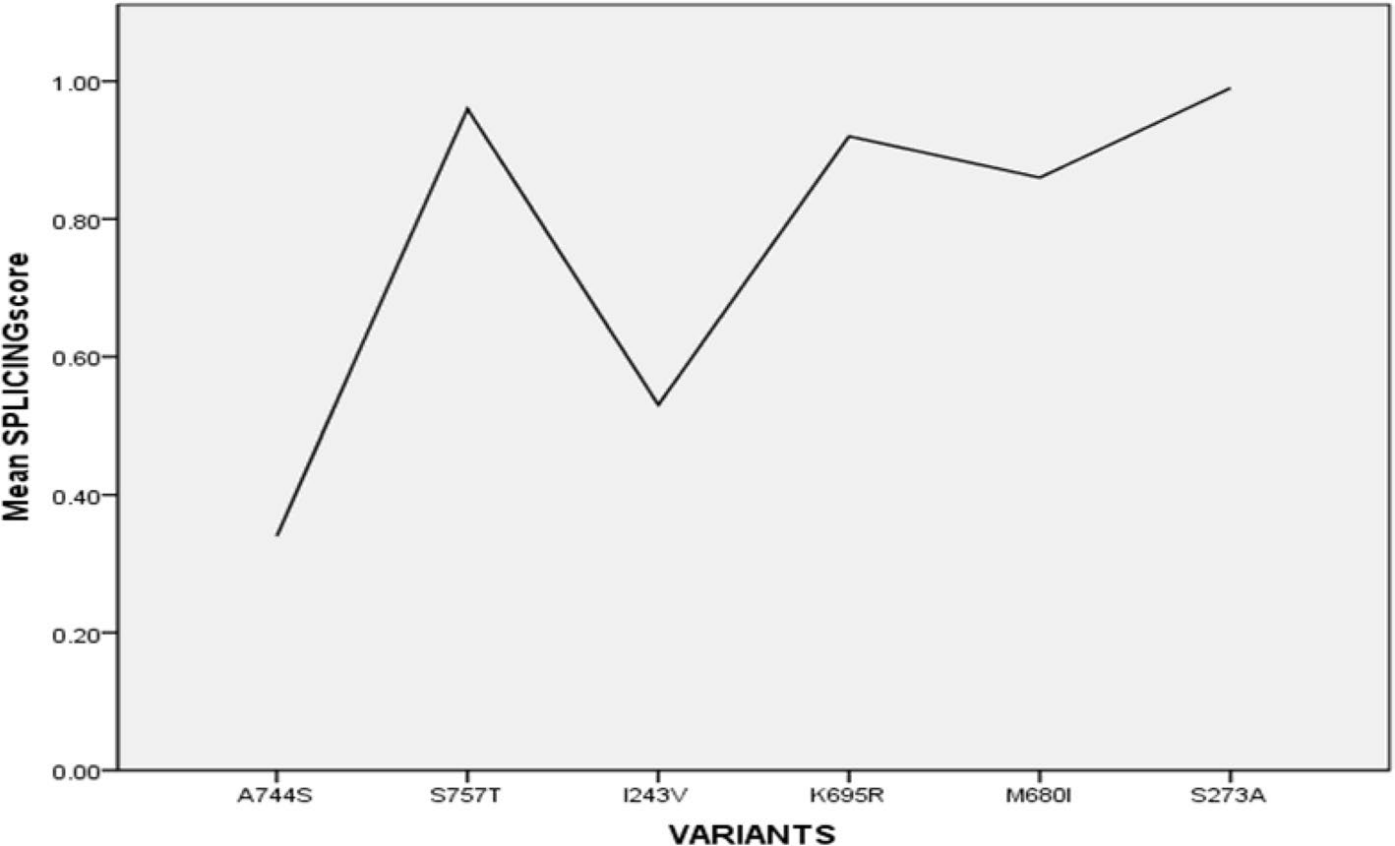
Predicted alternative splicing score for retrieved variants

Using MutationTaster and NNsplice softwares to compute the potential alternative splicing events which occur due to gain or loss of new donor or receptor site, S757T, S273A, and M680I recorded high scores (Fig. 6 and Table 3).

**Table 3:** Alternative splicing events due to some retrieved variants

AS events	Score	Exon-intron border
A757T		
Acc gained	0.96	accc|**C**TGG
K695R		
Donor gained	0.92	ATGA|**g**gga
M680I		
Donor gained	0.86	GGAA|cat**a**
S273A (main effect; loss of B30.2/SPRY domain)		
Donor gained	0.99	TCTG|gac**a**

## Discussion

The MEFV gene is located on chromosome 16p.13.3 producing a protein called pyrin or marenostrin which is implemented in familial Mediterranean fever (FMF) phenotype. FMF phenotypic analysis showed that most of severe FMF patients have variants located on the C-terminal B30.2/SPRY domain, and this domain was known as the hot spot of protein-protein interaction region (PPI) [21]. Therefore, the 13 variants located in B30.2/SPRY domain were retrieved from our previous study [10] to analyze its characteristics and probable pathogenicity. In the current study, annotation of B30.2/SPRY domain surface showed remarkable hydrophobic patches and grooves that might be used as surface binding sites for other proteins or small molecules, where L710, V726, A744, and S757 were localized on the wide hydrophobic grooves and S650, K671, S703, M694, S695, and K712 were localized on the edge of the hydrophobic cavities. It is obvious that the surface structure of pyrin is prone to be of high hydrophobicity and low polarity helping it to develop a high potential binding surface activity.

According to our previous published paper [10], most of the detected variants were located on B30.2/SPRY domain. Furthermore, all the registered crystallography 3D images were of B30.2/SPRY domain only; therefore, the current analysis was limited to B30.2/SPRY variant-depended computational algorithms.

In the huge genomic data era, it is worth to search about the mechanism of the pathogenicity of variants and correlate it with relevant diseases; this may obviously be a key player to define the precise clinical phenotype and consequently define the potential preventive or treatment drugs [22].

The study of the behavior of wild and mutant proteins via defining the distance between their physiochemical properties is a very helpful strategy to identify which region, domain, and variants are involved in the disease pathogenicity.

Wild pyrin is speculated to inhibit excess releasing of caspase-1 in the cytosol of the mononucleated cell and hence induced the inhibition of the inflammatory response. While the mutant pyrin is associated with decrease in the efficacy of that inhibitory function of Casp-1, and hence the pyrin is considered as an inhibitor of the proteolytic activation of caspase-1 [23, 24].

In the current study, the interface pockets among B30.2/SPRY domain of pyrin and the reported relevant reactants (ASC, CASP-1, ULK1, RHOA, NLRP1, NLRP3, IL18, and immature IL1b) was studied by the prism and visualized by discovery studio. Manual annotation and analysis of the retrieved variants within these PPI interface pockets showed that the specific variants are required to the protein-protein affinity and bonding. S650, S703, L710, K712, V726, and A744 are essential to form the multiprotein inflammasome complex containing pyrin according to our analysis.

In the current study, we aimed to explore the physiochemical surface and the characteristics of the molecular mechanics behavior of B30.2/SPRY and Casp-1/p20. We selected Casp1/P20 to study its binding with B30.2/SPRY without other reactants because it showed interface area wider than Casp1/P10. We identified that the interface pocket atoms between B30.2/SPRY and Casp-1 were most stable (low binding energy) than the binding energy value of B30.2/SPRY with ASC, Ulk1-LIR motif, RhoA, Nlrp1-LRR domain, Nlrp3, and IL18 and predicted a 3D of immature IL1b.

Approximately two thirds of all the FMF-associated variants clustered in B30.2/SPRY domain of pyrin. The 3D crystallographic image showed a shallow cavity covered mainly with hydrophobic amino acids which expected to contribute to the ligand-pyrin complexes by hydrophobic contacts [25]. The current results are in consistency with Weinert et al. [25] results, where a lot of hydrophobic patches, cavities, and grooves were found by computational tools, and these hydrophobic patches have significant binding tendency.

Using the PRISM server to predict the probable interface pocket atoms among B30.2/SPRY domain of Pyrin and six of its relevant interactors, p20 domain of Casp-1 protein had the lowest binding energy with B30.2/SPRY domain of pyrin. Accordingly, we extended the bioinformatics analysis on B30.2/SPRY-Casp1/p20 by computing the molecular mechanics (MM).

MM results showed that the wild B30.2/SPRY-Casp1/p20 complex had less value of binding energy (∆G) and dissociation constant (kd) than the mutant B30.2/SPRY-Casp1/p20, and hence the dynamic disequilibrium occurred towards the elevated quantity of the mutant B30.2/SPRY-Casp1/p20, and low quantity of free casp-1 in cytosol. These results agree with the findings of previous investigators [23, 24].

A novel pathway was previously reported that mutant pyrin enhances inflammation susceptibility via the hyperactivation of NF-kB, where Chae et al. [20] found that the susceptibility of the FMF-associated B30.2 pyrin variants to cleavage by Casp-1 were more than the wild-type pyrin producing cleaved N-terminal pyrin called N330, and the N330 had experimental evidence in both degradation of IkB-a (NF-kB suppressor) and increasing NF-kB nucleolization.

This novel pathway was based on the observation that the mutant B30.2 increased the potential to bind with casp1 than the wild type. The molecular basis and mechanism of action is not known yet. In the current study, we found that the interface binding of B30.2/SPRY domain with p20 of Casp1 have a larger binding surface than p10 of Casp1, and the molecular mechanics analysis of B30.2/SPRY-p20 complex harboring Lys671Met, Ser703Ile, and Ala744Ser variants showed that these variants led to an increase in the binding and stability of B30.2/SPRY-casp1/p20 complex.

The evident data of a novel mechanism of ASC-independent on the activation of NF-kB was reported by Chae et al. [20] which coincides with our molecular mechanics analysis of B30.2/SPRY-casp1/p20 complex. Certain patterns of the mutant B30.2/SPRY increased the potential to bind with Casp1-p20 than the wild type was observed. The modified predictive model was produced in the current study showing in what way the dynamic disequilibrium of B30.2/SPRY-casp1/p20 complex lead to the hyperactivation of NF-kB transcription factor and the increasing of IkB-a degradation. Furthermore, this modified predictive model could interpret the applicable rationality molecular reasons of the colchicine responsive spectrum and the increase of cytokines especially IL1b and IL18 in FMF patients.

IkB-a is known for its mechanism to suppress NF-kB entrance to the nucleus through masking the nuclear localization signal (NLS) of NF-kB. Haddad 2009 and Chae et al [20, 26] found that colchicine inhibits NF-κB hyperactivation via its role in IkB-a degradation suppression. Therefore, high dosage colchicine treatment might be effective with the high degradation of IkB-a.

The current global results of MM scores for interface residues (Ser744, Pro710, Ile703, Ala726, and Met671) of the B30.2/SPRY-casp1/p20 complex showed that mutant patterns of these residues could be classified as severity relevant variants. The severity impact for these variants is partially consistent with what obtained by Arakelov et al. [27].

## Conclusion

The current study predicted the dynamic disequilibrium of B30.2/SPRY-casp1/p20 complex phenomenon. Furthermore, this study highlights the specific biochemical markers that could be useful to adjust the colchicine dose, including Pyrin-N330, Casp1-p20, nuclear free NF-kB, 30KDa-IkB-a, IL1-b, and IL18. Our analysis also found a potential high score for the alternative splicing resulting from S757T, K695R, M680I, and S273A variants. Our main research limits were (1) 2wl1 PDB file to B30.2/SPRY domain, (2) 13 variants were previously published by us, and (3) B30.2/SPRY-casp1/p20 interface pocket for calculate MM scores. A further study is recommended to validate our modified predictive model.

## Data Availability

All the datasets used and/or analyzed during the current study are available from the first name author (Alaaeldin G. Fayez) on reasonable request.

## Abbreviations

FMF: Familial Mediterranean fever
PPI: protein–protein interaction
MM: Molecular mechanics
SAS: Solvent accessibility surface
RMSD: Root mean square deviation
